# Enhanced Accuracy of Continuous Glucose Monitoring during Exercise through Physical Activity Tracking Integration

**DOI:** 10.3390/s19173757

**Published:** 2019-08-30

**Authors:** Alejandro José Laguna Sanz, José Luis Díez, Marga Giménez, Jorge Bondia

**Affiliations:** 1 Centro de Investigación Biomédica en Red de Diabetes y Enfermedades Metabólicas Asociadas (CIBERDEM), Instituto de Salud Carlos III, 28029 Madrid, Spain; 2 Institut Universitari d’Automàtica i Informàtica Industrial, Universitat Politècnica de València, Camino de Vera, s/n, 46022 València, Spain; 3 Diabetes Unit, Endocrinology and Nutrition Department, Hospital Clínic Universitari, IDIBAPS (Institut d’investigacions Biomèdiques August Pi i Sunyer), 08036 Barcelona, Spain

**Keywords:** continuous glucose monitoring, sensor accuracy, exercise monitoring

## Abstract

Current Continuous Glucose Monitors (CGM) exhibit increased estimation error during periods of aerobic physical activity. The use of readily-available exercise monitoring devices opens new possibilities for accuracy enhancement during these periods. The viability of an array of physical activity signals provided by three different wearable devices was considered. Linear regression models were used in this work to evaluate the correction capabilities of each of the wearable signals and propose a model for CGM correction during exercise. A simple two-input model can reduce CGM error during physical activity (17.46% vs. 13.8%, *p* < 0.005) to the magnitude of the baseline error level (13.61%). The CGM error is not worsened in periods without physical activity. The signals identified as optimal inputs for the model are “Mets” (Metabolic Equivalent of Tasks) from the Fitbit Charge HR device, which is a normalized measurement of energy expenditure, and the skin temperature reading provided by the Microsoft Band 2 device. A simpler one-input model using only “Mets” is also viable for a more immediate implementation of this correction into market devices.

## 1. Introduction

Diabetes Mellitus is a disease characterized by a dysregulation of the natural homeostasis of glucose concentration in the body. It has a projected prevalence of 9.9% of the global population by 2045 (629 million people affected), and the estimated yearly cost in healthcare expenditure by the same year is 776 billion USD [1]. Approximately 10% of the people that have diabetes suffer from type 1 diabetes, which has a very early onset, usually manifesting during childhood or adolescence, and requires intensive supervision and treatment of the disease. Type 1 Diabetes (T1D) is defined by the inability of the beta cells of the pancreas to produce insulin in the bloodstream partially or totally, which leads to abnormally high levels of blood glucose. This problem worsens during periods of meal ingestion, in which glucose is poured into the bloodstream through the gut, or during physical activity or stress, which alter uptake or production of glucose in different parts of the body.

An Artificial Pancreas (AP) is a device that feeds continuous glucose measurements into an insulin pump in real time and continuously adjusts insulin dosage delivered to the patient. It is one the most promising solutions to the complications of T1D, and several prototypes have been under development over the last few years, including commercially-available [2] devices. Physical activity has been linked to changes in glucose trends and variability in patients with diabetes [3,4]. The accuracy of glucose measurement is of great importance for the correct performance of AP devices, since it drives the decision making of the algorithms behind it. The accuracy of Continuous Glucose Monitoring (CGM) devices has been observed to be affected during exercise periods. Taleb et al. [5] showed that both Dexcom G4 and Medtronic Enlite devices’ accuracy dropped during aerobic physical activity when compared to reference glucose measurements. Increased CGM error has been found consistently during aerobic exercise, even among the more recently distributed devices in the market [6]. Biagi et al. [7] showed that the accuracy of Medtronic Enlite 2 devices dropped both during aerobic and anaerobic exercise, but only the results during aerobic exercise were significantly different. This conclusion has also been reported in the literature before [5,8], which suggests an underlying problem with the mechanism of glucose estimation in the subcutaneous tissue during periods of physical activity.

Exercise monitoring is widespread today in most parts of the world, with many different devices designed and marketed to provide estimation of the intensity, type, and duration of physical activity. Integration of all available signals from wearable physical activity monitors (wearables) remains an open issue [9], and many of the wearable signals are only able to be extracted as processed variables. The use of wearable devices to improve T1D management has been reviewed before [10], and many studies have been conducted by adding different isolated wearable variables into AP controller algorithms [11], supervision algorithms [12], or prediction and classification algorithms [13,14]. Turksoy [15] studied the influence of wearable signals (named biometric variables in the reference) in the context of their possible implementation in an artificial pancreas system, showing that the amount of information carried by each signal changes depending on the type of exercise performed and placing special importance on the estimation of total energy spent by the physical activity.

The motivation for this work arises from the possibility of compensating the greater estimation error shown during exercise periods. Our working hypothesis is that exercise monitoring devices could provide information to a CGM device that allows for a real-time correction of the shift in glucose estimation during physical activity. The work presented in this paper analyzes the influence of the different signals provided by three different wearables (Fitbit Charge HR, Microsoft Band 2, and Polar HR) on the accuracy of a CGM device during aerobic exercise. Each wearable signal is critically selected or discarded as input for a multiple regression model designed to compensate the measurement error induced by the exercise session. The final regression model improves the accuracy of the CGM signal significantly during the exercise period, and baseline accuracy is maintained during the resting period. It is shown here that only two wearable signals are necessary to enhance the CGM measurements, and that redundancy of sensors only marginally increases the accuracy of the CGM estimations.

This paper is structured as follows. First, a breakdown of the methods, models, protocol, and datasets used is provided, including a brief description of all the devices employed in the study. Secondly, the results of the validation of the model utilized will be provided, along with the critical selection of signals to be used in the model. Then, a critical discussion of the results will be exposed, followed by the final conclusions.

## 2. Materials and Methods

### 2.1. Patients

The dataset used here was obtained from a longitudinal, prospective, interventional study with the goal of analyzing the performance of an exercise-challenged closed-loop controller in T1D people [16]. A total of six participants were enrolled at the Clinic University Hospital of Barcelona. The protocol was approved by the Ethics Committee of the hospital. Criteria for eligibility were: (1) age between 18 and 60 years old, (2) Body Mass Index (BMI) between 18 and 30 kg/m${}^{2}$, (3) Glycated Hemoglobin A1c (HbA1C) between 6.0% and 8.5%, and (4) use of Continuous Subcutaneous Insulin Infusion (CSII) for at least six months. Exclusion criteria included: (1) pregnancy, (2) use of experimental drug or devices in the past 30 days, (3) onset of progressive fatal diseases, (4) hypoglycemia unawareness, (5) drug or alcohol abuse, and (6) other systematic diseases other than T1D, including hepatic, neurological, and endocrine-related illnesses. A summary of the patient demographics is shown in Table 1.

Each participant underwent three aerobic and three anaerobic exercise tests. The order of the type of exercise was randomized, but the same type was carried out for three consecutive trials. The subjects used Paradigm Veo^®^ insulin pumps, and two Enlite-2^®^ (Medtronic Minimed, Northridge, CA, USA) CGM sensors were inserted in different parts of the abdomen the day before the trial by the subjects. Plasma glucose (PG) as a reference value was measured using the YSI 2300 Stat Plus Glucose Analyzer (YSI Incorporated Life Sciences, Yellow Springs, OH, USA), with a frequency of 15 min. The exercise schedules were as follows:

- Aerobic routine: Patients exercised doing three bouts of 15 min on a cycloergometer at 60% of the patients maximum capacity with five minutes of rest between them.
- Anaerobic (resistance) routine: Patients exercised doing five bouts of eight repetitions of four different exercise sets of 15 min at 70% of the patients’ maximum capacity with 90 s of rest between sets.

The exercise intensity was converted into units of heart rate, which was different for each patient and calculated as follows:

(1) $$HR_{i}^{\left(ex\right)}=\frac{Int}{100}\cdot \left(HR_{i}^{\left(max\right)}-HR_{i}^{\left(0\right)}\right)+HR_{i}^{\left(0\right)}$$

where $HR_{i}^{\left(ex\right)}$ is the intensity of the exercise in terms of heart rate for patient *i*, Int stands for the percentage intensity of the exercise (60 for aerobic exercise, 70 for anaerobic), and $HR_{i}^{\left(max\right)}$ stands for the maximum heart rate of patient *i*, defined as $226-age_{i}$ for women, and $220-age_{i}$ for men, where $age_{i}$ is the age of patient *i* in years. Lastly, $HR_{i}^{\left(0\right)}$ is the resting heart rate of patient *i* as measured at the beginning of the experiment.

### 2.2. Exercise Monitoring Devices

Off-the-shelf physical activity monitors were used to obtain the different biometric variables registered in the study. Patients wore three different devices simultaneously:

- Fitbit Charge HR™(Fitbit, San Francisco, CA, USA): a physical activity monitor that tracks several exercise signals: (1) heart rate, (2) steps, (3) floor level, (4) Metabolic equivalents (METs), and (5) calories burned.
- Microsoft^®^ Band 2 (Microsoft, Redmond, WA, USA): a multipurpose device that tracks physiological variables such as: (1) heart rate, (2) steps, (3) galvanic skin response, (4) skin temperature and (5) movements.
- Polar heart rate monitor, Model RCX3^®^ (Polar, Kempele, Finland): a commercially-established heart rate monitor.

The above listed variables were recorded with a sample rate of one minute and downloaded at the end of each trial. Each device provided a different number of signals, which will be listed next, for a total of 11 signals. The nomenclature of each signal needs to be defined in advance, since they will be referred to using this notation from here on. For each signal, a three-letter code was defined representative of the nature of that signal. The first letter of the triplet was the initial letter of the device that provided that signal, and the second and third letters were an abbreviation of the name of the signal itself. For example, the signal named **FHR** stands for **F** from the **F**itbit Device and **HR** from the **H**eart **R**ate signal.

1. **FHR**: **F**itbit **H**eart **R**ate; measured in beats per minute; can be used to estimate exercise intensity.
2. **FST**: **F**itbit **ST**eps; the number of steps walked.
3. **FLV**: **F**itbit **L**e**V**el; the number of floors of stairs climbed.
4. **FME**: **F**itbit **ME**ts; the metabolic equivalent of tasks; an estimation of exercise intensity normalized for each patient.
5. **FCA**: **F**itbit **CA**lories; calories burned.
6. **MHR**: **M**icrosoft Band **H**eart **R**ate.
7. **MTM**: **M**icrosoft Band **T**e**M**perature; skin temperature.
8. **MGS**: **M**icrosoft Band **G**alvanic **S**kin **R**esponse; an estimation of the skin electrodermal activity, also known as the conductance level of the skin.
9. **MST**: **M**icrosoft Band **ST**eps; number of steps walked.
10. **MMO**: **M**icrosoft Band **MO**vements; accumulated movement magnitude registered by the accelerometer.
11. **PHR**: **P**olar **H**eart **R**ate.

### 2.3. Data Filtering and Pre-Processing

Thirty six trials were completed, and data were collected for all of them. In order to align CGM and YSI data, CGM data were linearly interpolated and rounded to one sample per minute. Following the rationale described in [7], data arrays corresponding to either CGM or YSI malfunction, error outliers, or more than 30% of data samples flagged as faulty were discarded. A total of 665 sampled pairs were available, distributed among 27 streams of data out of the 36 corresponding to paired errors of CGM vs. YSI following the rule:

(2) $$E_{i}\left(k\right)=CGM_{i}\left(k\right)-PG_{i}\left(k\right)$$

where $E_{i}\left(k\right)$ represents the CGM estimation error for data stream *i* at timestamp *k*. Similarly, $CGM_{i}\left(k\right)$ and $PG_{i}\left(k\right)$ are the CGM data and plasma glucose data measured with the YSI. Each data stream *i* is a time series corresponding to a particular experiment and CGM signal.

CGM accuracy was evaluated using the Mean Absolute Relative Deviation (MARD), calculated as:

(3) $$MARD_{i}=\frac{\sum_{k=1}^{n}\frac{\left|E_{i}\left(k\right)\right|}{PG_{i}\left(k\right)}}{n}$$

where $MARD_{i}$ is the average percent error of the data stream *i* and *n* is the total number of samples available for *i*.

Wearable data were pre-processed by removing the *baseline* value of each wearable signal and trial from the signal itself. The *baseline* of each signal was calculated as the median value of that particular variable for each data stream evaluated only in the resting period. In an out-of-clinic environment, this value is trivial to obtain since it would be the equilibrium value of the signal during resting or sleeping periods.

The availability of the different wearable variables, defined as the complementary value of the percentage of missing samples, varied from signal to signal. In Table 2, the availability of each one of the signals obtained from the wearables is provided.

### 2.4. Data Analysis

#### 2.4.1. Model Fit

Linear regression models were used to fit the CGM error data, using only the wearable signals as inputs. The intercept of the model was forced to be zero in order to better compensate only the error observed in the exercise period. The linear regression model follows the following equation:

(4) E=Θ·p

(5) Θ=[FHR,FST,FLV,FME,FCA,MHR,MTM,MGS,MST,MMO,PHR];

where p is the vector of regression parameters, of a length equal to the number of wearable signals used. Each of the components of Θ, corresponding to each of the wearable signals, is a column vector of length *n*. Finally, the new enhanced CGM (eCGM) estimations, corrected using the new estimated error, is calculated as follows:

(6) $$eCGM_{i}=CGM_{i}-E_{i}$$

where $eCGM_{i}$ represents the enhanced CMG values for data stream *i*, $CGM_{i}$ are the original sensor glucose estimations, and $E_{i}$ are the output of the linear regression above.

#### 2.4.2. Exercise Signal Selection

In order to determine which of the wearable signals in the study were more relevant for our purposes, a backwards elimination of variables approach was followed, in which variables were sequentially discarded as inputs to the correction model, based on:

- Lack of availability of synchronous data to the CGM-YSI available pairs.
- Lack of representation of the type of physical activity performed in the study: cycloergometer aerobic exercise.
- High correlation between wearable signals. A high correlation between two signals implies that the amount of information carried by each one of those signals is similar; thus, one of them can be eliminated without hindering the predictive capabilities of the model.
- MARD improvement evaluated in the validation sets (see Section 2.4.3 below). A wearable was removed from the input set if the MARD for the validation sets (both in the exercise period and in global terms) was improved by removing that wearable signal alone.

If a variable complied with the above rules, it was removed from the inputs of the multiple regression model, and the model was fit again to the data and the outputs re-evaluated, until no improvement of the error was achieved by removing any of the inputs.

#### 2.4.3. Cross-Validation

The predictive power of the model was validated by performing a non-exhaustive leave nine-out cross-validation of the model. Out of the set of 27 streams of data, two thirds (i.e., 18 randomly-chosen streams out of 27) were selected as a fitting set, and the remaining data were used to validate the fitted model. Model accuracy was evaluated on the validation set, the results stored, and the process was repeated, randomly selecting new fitting and validation sets. This process was repeated 20 times. All the accuracy metrics shown onward were those averaging the results throughout all the 20 validation sets of nine streams of data.

## 3. Results

### 3.1. Wearable Selection

**Figure 1:**
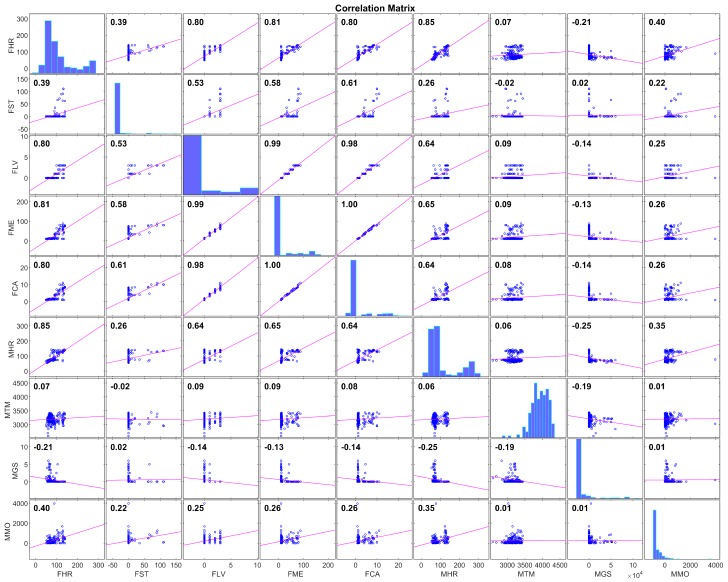
Correlation matrix of the wearable signals from the subset of valid variables selected. Note that the quantization of some of the variables renders point density in some of the subfigures very different from others. For example, most of FST values are located around zero, which makes linear regression plots look like the line does not fit the data, but this is not the case.

Any wearable with 30% or more samples not usable to fit the model, such as missing samples, disconnections, or out of bounds samples, was discarded from the fit. Following the backwards elimination philosophy previously detailed, the first two wearable signals discarded from the model fit were MST and PHR due to their low availability in Table 2, leaving the remaining Θ matrix to fit the model:

(7) Θ=[FHR,FST,FLV,FME,FCA,MHR,MTM,MGS,MMO];

Next, the correlation between the remaining signals was computed, with the results in Figure 1.

The correlations between FLV, FME, and FCA (all derived from the accelerometer sensor) showed that those three variables carried essentially the same information. FME was deemed a more relevant variable than the other two due to its continuous nature (FLV is discrete by definition) and the fact that it was normalized by the baseline energy consumption of each patient, which suggests that a linear model using this variable as an input would require less individualization than one using the other variables. Thus, it was decided to discard FLV and FCA from the model fit.

Similarly, both FHR and MHR were highly correlated (R=0.85), which is coherent with the nature of those variables, since both of them estimated the same physiological value. It was decided that FHR was more relevant than MHR for the linear model due to its higher availability, as shown in Table 2. Thus, MHR was discarded as an input to the model.

**Figure 2:**
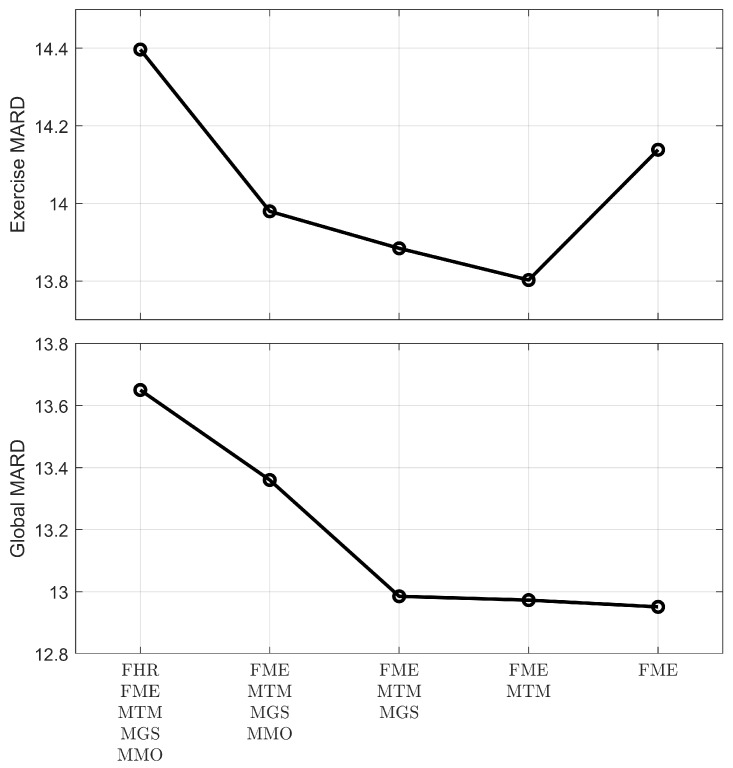
Sequence of MARD values for the exercise period (**top panel**) and for the whole trial (**bottom panel**), as each signal was removed from the model.

Finally, FST was deemed irrelevant for the type of exercise performed in the trials. FST estimates the intensity of physical activity and quantifies that value in the number of steps walked by the patient. The physical activity from this study (cycloergometer) rendered the patient static for most of the exercising period, which would result in inaccurate estimations of the steps. Furthermore, the histogram of the signal FST in Figure 1 shows that most of the FST values laid very close to zero. Removing FST from the inputs list left the input parameter array as:

(8) Θ=[FHR,FME,MTM,MGS,MMO];

Using the five remaining signals as inputs, the linear model was fit, and the results of the MARD improvement in the validation sets were recorded. A single input was removed one at a time, the model’s performance recorded, and the cycle repeated, removing another input and using the others. The input elimination that further worsened the MARD in the exercise period, or the one that most worsened the MARD for the whole trial period in case of equal exercise MARD values, was permanently eliminated. The sequence of eliminations and the results on the MARD reduction are shown in Figure 2.

The model improved plasma glucose estimations as inputs were removed from the system, especially when looking at the whole period of monitoring (global MARD). However, the global MARD reduction from removing the two last signals, MTM and MGS, was minimal. MARD in the exercise period was also reduced by removing the same signals except when removing MTM. Thus, the final selected list of inputs for the model is:

(9) Θ=[FME,MTM];

### 3.2. Error Grid Analysis and Model Performance

The final order of the model was two, since only two signals were used as the input. The average values of coefficients for the model fit are shown in Table 3, along with the parameters of the model, fitting *all* available data and not separating any for validations.

MARD results for the proposed model are shown in Table 4, along with the Coefficient of Variation (CV; defined as CV =100·SD(CGM)/mean(CGM), where SD(•) stands for the standard deviation and mean(•) stands for the mean value) to evaluate glycemic variability.

MARD during the exercise period was reduced drastically and significantly, from 17.46% to 13.8%, which was much closer to the average MARD of the original CGM of 13.61%. The total MARD (CGM error in the whole dataset, not separating resting and exercise periods) was reduced slightly to 12.97%, and the error during the resting period was increased marginally to 12.88%, but both figures were not significantly different than those presented by the original CGM.

**Figure 3:**
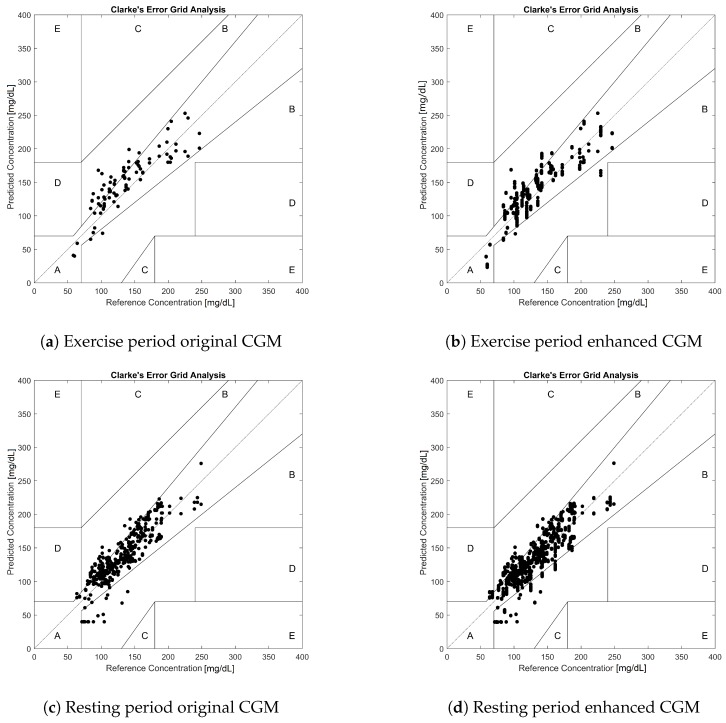
Clarke error grid analysis for the wearable-enhanced CGM data. (**a**) Subset of CGM-PG pairs *before* enhancement during the physical activity period. (**b**) Subset of CGM-PG pairs *after* enhancement with exercise monitoring devices during the physical activity period. (**c**) Subset of CGM-PG pairs *before* enhancement during the resting period. (**d**) Subset of CGM-PG pairs *after* enhancement with exercise monitoring devices during the resting period.

The validation data were evaluated using different acceptability criteria. The Clarke error grid analysis of the data [17] is depicted in Figure 3. Note that since 20 iterations of the fit-validate cycle were performed, there were several data points corresponding to the same PG value, displaying vertical patterns in the eCGM subplots. This also means that the point density of the eCGM subplots was much higher than those of the original CGM.

The numeric values of the results for the Clarke EGA can be seen in Table 5. The results showed a displacement towards the A zone, especially during exercise, as a response to the proposed correction.

**Figure 4:**
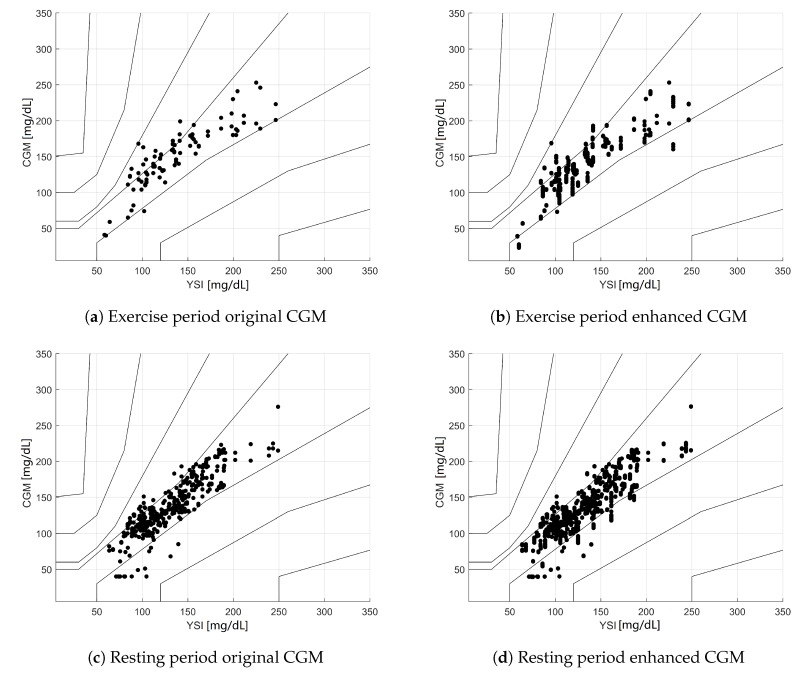
Figure 4. Parkes error grid analysis for the wearable-enhanced CGM estimations. (**a**) Subset of CGM-YSI pairs *before* enhancement during the physical activity period. (**b**) Subset of CGM-YSI pairs *after* enhancement with exercise monitoring devices during the physical activity period. (**c**) Subset of CGM-YSI pairs *before* enhancement during the resting period. (**d**) Subset of CGM-YSI pairs *after* enhancement with exercise monitoring devices during the resting period.

In addition to the Clarke EGA analysis, Figure 4 shows the results of the Parkes EGA [18], which classifies the same pairs of data into different zones, with the advantage of showing more continuous transitions between adjacent zones in the PG-CGM plane. Table 6 shows the numeric results for the same EGA analysis.

**Figure 5:**
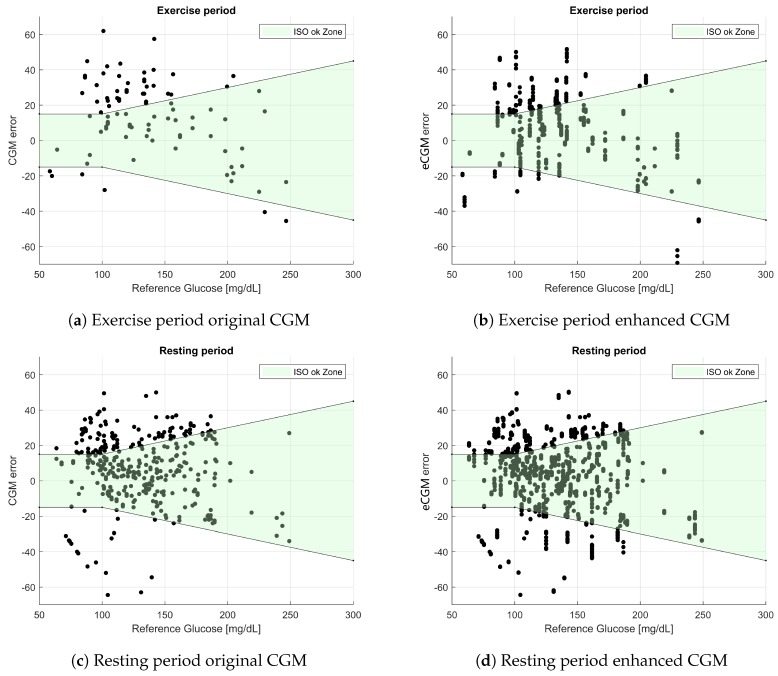
ISO acceptable region for the wearable-enhanced CGM errors. (**a**) Subset of error-YSI pairs *before* enhancement during the physical activity period. (**b**) Subset of error-YSI pairs *after* enhancement with exercise monitoring devices during the physical activity period. (**c**) Subset of error-YSI pairs *before* enhancement during the resting period. (**d**) Subset of error-YSI pairs *after* enhancement with exercise monitoring devices during the resting period.

In Figure 5, the results for the ISO standard defined in [19] are shown. The ISO classification had only two zones, separating the CGM-PG pairs into “OK” estimations and “not OK” estimations. The total number of samples in each category is reported in Table 7.

## 4. Discussion

The first outcome that can be extracted from the results is that further integration of physiological signals in CGM devices is desired. CGM devices use algorithmic rules to fit the chemical measurements of the subcutaneous probe into relatively accurate plasma glucose values. If the data extracted from wearable devices were to be integrated in those algorithms, glucose estimation would benefit greatly.

The proposed linear regression model (composed of only two parameters) achieved a reduction in the MARD during aerobic exercise that practically nullified the influence of the exercise on the CGM estimation error. Indeed, the MARD was significantly reduced from 17.46% to 13.8%, much closer to the global average MARD value for the original CGM readings of 13.61%. This achievement was the main reason to choose a regression model for error compensation, which validated the starting hypothesis.

The regression model presented here differed from other studies involving wearables in the AP (see [11,12]) in the fact that the work presented here supposed a more general result, its application being not only limited to closed loop studies; the work presented here can be used to provide better estimations to an AP device, but it is not limited to that. It can also be used to provide simply more accurate continuous glucose readings to people with diabetes regardless of whether or not they are using an AP device or undergoing CSII treatment.

The Clarke EGA shown in Figure 3 indicated very encouraging results for the exercise period; while the original CGM already had 100% of the samples in the A + B zone, eCGM showed a shift of the data points towards the A zone for the exercise period (72.4% → 85.7%). However, Clarke’s analysis also showed a very small rise of points in Zone D during the rest period (0.2% → 0.6%), while no points were present in Zone C. This is a consequence of the discontinuity in the zones defined for Clarke’s EGA, where Zone D can be accessed directly from Zone A or B. This is one of the main criticisms [18] against Clarke’s EGA, and it was the reason that motivated the comparison with other analysis methodologies.

The results shown in Table 6 are even more positive, showing an increase of points in Zone A (79.2% → 89.2%), while showing no points in Zones C + D + E. ISO results from Table 7 also showed better results for eCGM than the original sensor, increasing the amount of “OK” samples 55.2% → 64.4% during the exercise period, as well as increasing the total samples in the ISO zone 66.6% → 68.8%

Figure 2 compares MARD between different proposed models of decreasing complexity, showing also increasing performance in the validation error. The difference in the global MARD values in the last two models was almost non-existent, while the MARD during the exercise phase of the study actually worsened. However, these differences were very small when compared to the MARD reduction achieved by utilizing any of those models versus not using any model to compensate for exercise (17.46% → 13.8% and 17.46% → 14.14%). It can be argued that using a model with only one parameter is preferable than the more complex option of a two-parameter model, especially when looking at the fact that both input signals came from different devices on the market: FME was provided by the Fitbit device, and MTM was extracted from the Microsoft device.

This work did not focus on the nature of the signals used in the model. Rather, the focus here was on the user-level data that can be easily extracted from a device, which translates into a more straightforward implementation of the model in portable devices. This implies that some of the signals that were estimated from each of the devices using hidden algorithms might not be reproducible from other raw data, e.g., FME was an energy expenditure estimation by the Fitbit device calculated from its sensors, which can differ from implementations in other devices. In a recent study comparing different research and consumer devices, an error range of 10% was found for heart rate and energy expenditure measurements [20].

It must be noted that the current approach has some drawbacks due to its intentional simplicity. First, while the CGM error was reduced significantly in the exercise period, this was achieved by means of a direct algebraic link to the signals of the wearable devices, which may transport noise from the wearable into the CGM, but that problem was not observed in our data. Additionally, it must be mentioned that more complex dynamic models might have further enhanced CGM accuracy, but the design of such models is beyond the scope of this work.

## 5. Conclusions

This paper showed that continuous glucose estimation can benefit from the integration of signals readily available from wearables. CGM estimation error was improved during the exercise period and was not significantly increased otherwise. The model proposed was composed of two parameters; however, a simpler model composed of only one parameter can also yield acceptable results, and the technology is readily available with off-the-self devices.

## Figures and Tables

**Table 1:** Participants’ characteristics. Data expressed as the mean ± standard deviation.

Number of Patients (Females)	6 (1)
Age (years)	36.7±8.9
HbA1c (%)	7.9±0.5
BMI (kg/m${}^{2}$)	24.6±1.0
Time with T1D (years)	25.2±12.7
Time with CSII (years)	4.8±1.7

**Table 2:** Wearable signals’ availability. **FHR**: **F**itbit **H**eart **R**ate; **FST**: **F**itbit **ST**eps; **FLV**: **F**itbit **L**e**V**el; **FME**: **F**itbit **ME**ts; **FCA**: **F**itbit **CA**lories; **MHR**: **M**icrosoft Band **H**eart **R**ate; **MTM**: **M**icrosoft Band **T**e**M**perature; **MGS**: **M**icrosoft Band **G**alvanic **S**kin **R**esponse; **MST**: **M**icrosoft Band **ST**eps; **MMO**: **M**icrosoft Band **MO**vements; **PHR**: **P**olar **H**eart **R**ate.

	FHR	FST	FLV	FME	FCA	MHR	MTM	MGS	MST	MMO	PHR
Availability (%)	96.4	100	100	100	100	78.7	73.3	78.7	14.7	73.3	55.3

**Table 3:** Proposed model parameters. $p_{FME}$ stands for the FME signal coefficient in p and $p_{MTM}$ the coefficient for the MTM signal in p.

Parameter	$p_{\mathrm{FME}}$	$p_{\mathrm{MTM}}$
Average parameter in cross-validation	0.33	−0.0017
Parameter fitting all data	0.297	−0.0053

**Table 4:** MARD and CV results for the selected model.

		Original CGM	Enhanced CGM	*p*-Value
MARD	Exercise period	17.46%	13.8%	<0.05
	Resting Period	12.75%	12.88%	0.75
	Total	13.61 %	12.97 %	0.4
CV		17%	16%	0.17

**Table 5:** Table of the results for the classification of the CGM samples in the different Clarke EGA zones. CGM-PG refers to paired CGM and PG data.

Zone			A	B	C	D	E
CGM-PG pair allocation (%)	Exercise Period	CGM	72.4	27.6	0	0	0
		eCGM	85.7	14.3	0	0	0
	Resting Period	CGM	85.6	14.2	0	0.2	0
		eCGM	84.3	15.1	0	0.6	0
	Total	CGM	82.6	17.2	0	0.2	0
		eCGM	84.6	15	0	0.4	0

**Table 6:** Table of results for the classification of the CGM samples in the different Parkes EGA zones.

Zone			A	B	C	D	E
CGM-PG pair allocation (%)	Exercise Period	CGM	79.2	20.8	0	0	0
		eCGM	89.2	10.8	0	0	0
	Resting Period	CGM	90.5	9.5	0	0	0
		eCGM	87.7	12.3	0	0	0
	Total	CGM	88	12	0	0	0
		eCGM	88	12	0	0	0

**Table 7:** Table of the results for the classification of the CGM samples within the bounds as defined in ISO 15197: 2013.

	ISO Valid Samples	
	Original CGM	Enhanced CGM
Exercise Period	55.23 %	64.4%
Resting Period	69.9%	70.1%
Total	66.6%	68.8%

## Abbreviations

The following abbreviations are used in this manuscript:

MARD	Median Absolute Relative Deviation
PG	Plasma Glucose
CGM	Continuous Glucose Monitor
eCGM	Enhanced Continuous Glucose Monitor
AP	Artificial Pancreas
T1D	Type 1 Diabetes
BMI	Body Mass Index
HBA1c	Glycated Hemoglobin (A1c)
CSII	Continuous Subcutaneous Insulin Infusion
CV	Coefficient of Variation
EGA	Error Grid Analysis
